# Epidemiological pattern of dengue fever in Afghanistan in the period 2021–22

**DOI:** 10.1093/eurpub/ckae116

**Published:** 2025-01-13

**Authors:** Mohamed M Tahoun, Mohammad O Mashal, Abdul Rahman Laiq, Abdul Wahid Amiri, Sherein T A Elnossery, Marwa Rashad Salem, Nelly S Hegazy, Sherif E Eldeeb, Fazal Elahi Alizai, Ehsanullah Misbah, Jamshed A Tanoli, Alaa H Abouzeid

**Affiliations:** Health Emergencies Department, World Health Organization Country Office, Kabul, Afghanistan; Department of Epidemiology, High Institute of Public Health, Alexandria University, Alexandria, Egypt; Health Emergencies Department, World Health Organization Country Office, Kabul, Afghanistan; Health Emergencies Department, World Health Organization Country Office, Kabul, Afghanistan; Health Emergencies Department, World Health Organization Country Office, Kabul, Afghanistan; World Health Organization Office for the Eastern Mediterranean Region, Cairo, Egypt; Public Health and Community Medicine Department, Faculty of Medicine, Cairo University, Cairo, Egypt; Public Health and Community Medicine, Faculty of Medicine, Helwan University, Cairo, Egypt; Community Medicine Research Department, National Research Centre, Cairo, Egypt; Monitoring and Evaluation and Health Information System, Ministry of Public Health, Kabul, Afghanistan; Monitoring and Evaluation and Health Information System, Ministry of Public Health, Kabul, Afghanistan; Health Emergencies Department, World Health Organization Country Office, Kabul, Afghanistan; Public Health and Community Medicine Department, Faculty of Medicine, Cairo University, Cairo, Egypt

## Abstract

Dengue fever is considered as an emerging disease in Afghanistan. Since the first outbreak was reported in 2019, other outbreaks have been reported in the following years. The current study aims to describe the epidemiological features and clinical manifestations of suspected and confirmed cases of dengue fever detected by the National Disease Surveillance and Response (NDSR) Department of the Ministry of Public Health (MoPH) during 2021 and 2022 to prevent further spread and minimize its impact on the country’s health system and on the limited number of health workers. Through a retrospective analysis of historical data related to suspected dengue fever cases in Afghanistan detected by the National Disease Surveillance and Response Department during 2021 and 2022, several variables were identified, including demographic characteristics, clinical features, clinical management, the outcome of infection, laboratory data, and epidemiological factors. All statistical analyses were developed using Microsoft 365 (Excel). The mean age of the 1977 reported suspected dengue fever cases was 30.4 ± 14.9 years, with males 70.7%. The epidemic curve showed a steep rise in cases in 2022, starting from week 39, with a peak reached in week 45, which was higher than that observed in 2021. The majority of cases (97.9%) were reported from Nangarhar Province, east of the country. Regarding symptoms, fever, headache, and muscle pain were expressed in nearly all cases. The reverse transcription polymerase chain reaction was positive in 379 cases out of 497 cases (76.3%). For the management of cases, 97.6% received antipyretics. Less than 5% of cases were admitted to health care units, with death reported in only two cases (case fatality rate of 0.1%). The number of suspected cases of dengue fever reported in Afghanistan was increasing. The trends for 2021 and 2022 followed almost the same pattern, with a higher peak in 2022. Understanding the epidemiological and clinical characteristics of dengue fever cases is fundamental for preparedness for upcoming seasons.

Key points

- The number of suspected dengue fever reported in Afghanistan was increasing.
- The trends for 2021 and 2022 followed almost the same pattern with a higher peak in 2022.
- Understanding the epidemiological and clinical characteristics of the dengue fever cases is fundamental for preparedness for upcoming seasons.
- The current study aims to describe and document the epidemiological features and clinical manifestations of suspected and confirmed cases of dengue fever to prevent further spread and minimize its impact on the country’s health system and on the limited number of health workers.

## Introduction

Over the past years, dengue fever has started to emerge on a rising scale globally, according to the World Health Organization (WHO) [1]. It is among the most significant infectious diseases, having a high disease burden and cycles of epidemics every 3–5 years [2]. The disability-adjusted life years (DALYS) of dengue in the Southeast Asian region were 372 DALYs per million per year [3].

Dengue is endemic in different countries in the Eastern Mediterranean region; however, it has been spreading to new areas in recent years. Outbreaks of dengue fever have emerged sporadically in Afghanistan. The first outbreak was reported in 2019 in the eastern region of Afghanistan, with only 15 cases [4]. However, in 2021, the disease had resurfaced, infecting 775 people and killing one in Nangarhar Province alone [3]. A new wave of dengue fever has been confirmed in Afghanistan, with a total of 346 cases reported since the beginning of 30 June 2023, from Nangarhar Province only, with one associated death. Of the 346 reported cases, 202 (58.4%) were females, and the majority (97.4%) were over five years of age [5].

Dengue epidemics tend to have seasonal patterns, with transmission often peaking during and after rainy seasons. In Afghanistan, the rainy season starts in September and ends in March each year, which provides a suitable environment for vector breeding in endemic areas. Dengue infection is usually asymptomatic in >50% of cases; alternatively, it can present as a flu-like illness, including headache, myalgia, and rash [6]. Therefore, knowledge of dengue's geographical distribution and burden is crucial. To date, there are no licensed vaccines or specific therapeutics for dengue [2]. The current study aims to describe the epidemiological features and clinical manifestations of suspected and confirmed cases of dengue fever detected by the National Disease Surveillance and Response (NDSR) Department of the Ministry of Public Health (MoPH) from 1 January 2021 till 31 December 2022 to prevent further spread and minimize its impact on the country’s health system and on the limited number of health workers.

## Methods

A retrospective analysis of historical data on dengue fever cases in Afghanistan from January 2021 to December 2022 was conducted using the NDSR dengue fever queue list (case-based data).

*The case definition used to collect these data in the queue list was as follows:*

*Suspected case:* any person with an acute onset of fever (>38°C) for 2–10 days with at least two of the following manifestations: severe headache, retro-orbital pain, myalgia or arthritis, and a positive tourniquet test [7].

*Confirmed case:* a suspected case was confirmed by the laboratory by one or more of the following:

- Isolation of the dengue virus from serum, plasma, leukocytes or autopsy samples.
- Demonstration of a four-fold or greater change in reciprocal IgG or IgM antibody titer to one or more dengue virus antigens in paired serum samples.
- Demonstration of dengue virus antigen in autopsy tissue by immunohistochemistry or immunofluorescence or in serum samples by enzyme immunoassay (EIA).
- Detection of viral genomic sequences in autopsy tissue, serum or CSF samples by polymerase chain reaction (PCR).
- Cases with the positive rapid diagnostic tests (RDTs) [8].

Since January 2022, the NDSR, with the support of the WHO country office, has maintained a queue list of data on both suspected (based on clinical features) and laboratory-confirmed dengue fever cases. This queue list was used for all analyses. These queue lists are kept in the format of Microsoft Excel workbooks for analysis by the data management officers at the NDSR. Several variables were identified in the database, including demographic characteristics (age, sex, and geographic location), clinical features (symptoms such as fever, headache, muscle pains, and hemorrhage), clinical management [receiving antibiotics, intravenous (IV) fluids and antipyretics], the outcome of infection, laboratory data and epidemiological factors (possible source of infection and travel history).

Categorical variables were expressed in numbers and percentages. Quantitative variables were expressed using the median and the interquartile range, mean and standard deviation. The overall cumulative incidence of dengue fever was calculated per 1000 population for Kabul, Nangarhar and Laghman provinces and their districts in the period from 2021 to 2022. All statistical analyses, including the epi curves, were developed using Microsoft 365 (Excel). The map for the distribution was developed using the geographic information system (GIS) ArcMap 10.5.1 software.

## Results

As shown in Table 1, the total number of reported cases of dengue fever in the period 2021–2022 was 1977, with 1398 males (70.7%). The mean age was 30.4 ± 14.9; a total of 527 cases (26.7%) were between the age groups 16 and 25 years, followed by 525 cases (26.6%) and 308 cases (15.6%) among the age groups 26–35 and 36–45 years, respectively. The lowest proportion of cases, comprising 45 cases (2.3%), pertained to individuals at the extremes of age, specifically those under five years old and those older than 75. Only 47 cases (2.4% of cases) reported travel history to the dengue-endemic country, Pakistan.

**Tables 1.** Sociodemographic characteristics and exposure history of dengue fever cases in the period from 2021 to 2022 (*n* = 1977)

Characteristics	*N*	%
Age		
0–5	37	1.9
6–15	270	13.7
16–25	527	26.7
26–35	525	26.6
36–45	308	15.6
46–55	184	9.3
56–65	92	4.7
66–75	26	1.3
>75	8	0.4
Sex		
Male	1398	70.7
Female	579	29.3
Province		
Nangarhar	1937	97.98
Kabul	39	1.97
Laghman	1	0.05
Travel history to an endemic country two weeks before the onset of the symptoms^a^	47	2.4

a The visited country was Pakistan.

Supplementary Fig. S1 shows geographical locations of the dengue fever cases in Afghanistan. A total of four provinces mainly in the east region of the country reported dengue fever cases during 2021 and 2022 outbreaks. The initial cases of the outbreak were detected and reported from Momand Dara district from where the cases were expanded to other districts of Nangahar and nearby provinces (Laghman, Kunar, and Kabul). Nangarhar Province accumulated 1937 cases, representing 97.98% of the total cases, with the highest incidence rate in the Momand Dara district, which was 6.1–25.1, followed by 3.1–6 in the Shinwar and Dur Baba districts. Less than 2% of cases were from the province of Kabul. However, all 39 cases (1.97%) from Kabul either came from the east region or had a travel history to endemic areas before showing symptoms.

**Figure 1.**
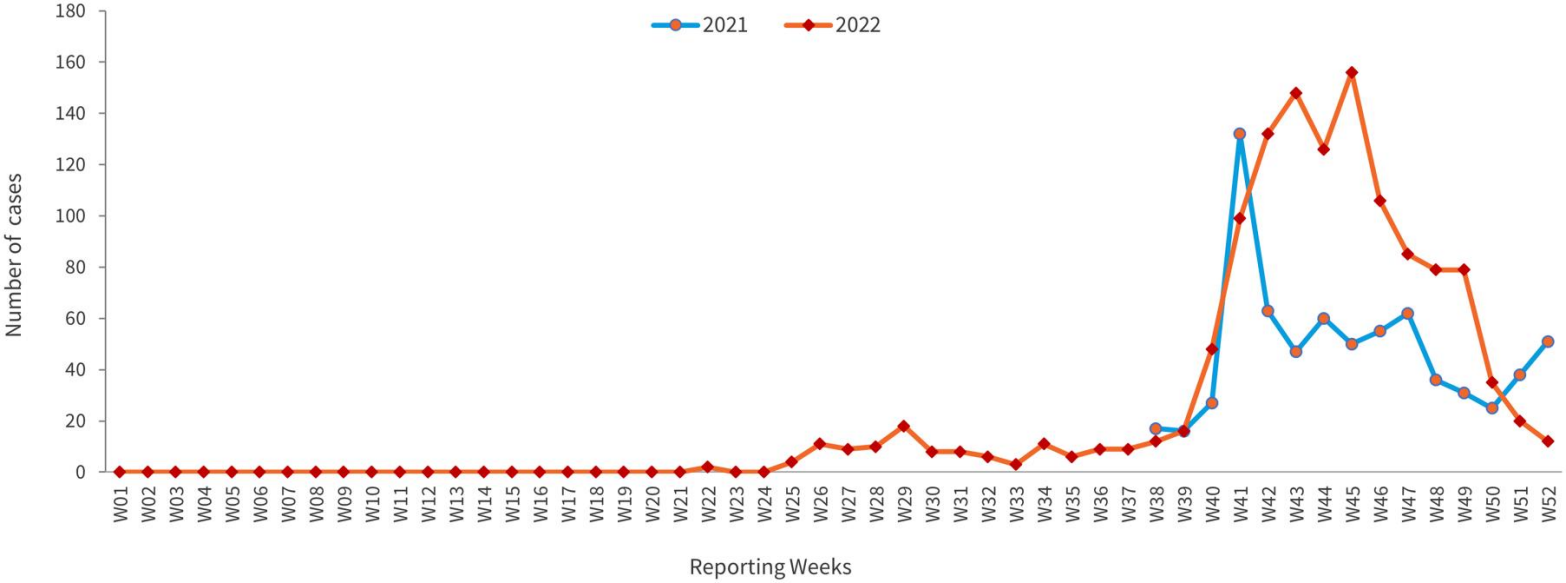
The trend of suspected dengue cases in Afghanistan over the years 2021–22.

Supplementary Fig. S2 demonstrates the epidemic curve of dengue cases in Afghanistan for the years 2021 and 2022. The curve indicates a steep rise in dengue cases during the fall season of both years, with specific peak periods. In 2021, the epidemic curve showed a notable increase in cases starting from week 38, corresponding to the end of September 2021. The peak of the epidemic occurred during week 41, around mid-October 2021. In 2022, a similar pattern was observed, with a steep rise in cases starting from week 38 (end of September 2022) and reaching a peak in week 45, which falls in mid-November 2022. Importantly, the peak in 2022 was noted to be higher than that in 2021, as shown in Fig. 1, which displays the trend of dengue cases in Afghanistan over the years 2021–22. This pattern suggests a seasonal trend in dengue cases, with a consistent increase during the fall rainy season in both years. The higher peak in 2022 indicates a potential escalation of dengue incidence compared to the previous year.

Table 2 displays the clinical manifestations of dengue cases in the period from 2021 to 2022. Regarding the symptoms, fever, headache and muscle pain were expressed in nearly all cases (96.4%, 94.9% and 94.8%, respectively). Additionally, 1359 cases (68.7%) reported nausea and vomiting. Severe dengue has not been reported; no cases have been reported with skin rash, severe bleeding, severe plasma leakage or severe organ involvement. Concerning the laboratory investigations, the RDT was positive in 1032 cases out of 1086 cases (95%). An ELISA IgM was positive in 125 cases out of 312 cases (40.1%), ELISA IgG was positive in 58 cases out of 319 cases (18.2%) and reverse transcription polymerase chain reaction (RT-PCR) was positive in 379 cases out of 497 cases (76.3%). The median duration of the collection date of samples post-infection was 4 days (IQR = 1), while the median duration until receiving the results was two days (IQR = 3).

**Table 2.** Clinical manifestations and laboratory findings of dengue cases in the period from 2021 to 2022

Variables	*N*	%
Symptoms and signs (*n* = 1977)		
Fever	1906	96.4
Headache	1876	94.9
Muscle pain	1874	94.8
Nausea and vomiting	1359	68.7
Laboratory investigations		
PCR^a^ (*n* = 497)		
+ve	379	76.3
ELISA^b^ IgG (*n* = 319)		
+ve	58	18.2
ELISA^c^ IgM (*n* = 312)		
+ve	125	40.1
Rapid diagnostic test (*n* = 1086)		
+ve	1032	95.0
Duration of collection date of samples post-infection (in days)		
Median (IQR)^d^	4 (1)	
Duration between sending to lab and results (in days)		
Median (IQR)	2 (3)	

a PCR: polymerase chain reaction.

b ELISA IgG: an enzyme-linked immunosorbent assay (ELISA) was employed to detect specific human immunoglobulin G.

c ELISA IgM: an enzyme-linked immunosorbent assay (ELISA) was employed to detect specific human immunoglobulin M.

d IQR: interquartile range.

For the management of cases, 1930 cases (97.6%) received antipyretics; antibiotics were prescribed to 1719 cases (86.9%), whereas 217 cases (10.9%) needed intravenous fluids. A total of 1881 cases (95.1%) were managed in outpatient services; while 94 cases (4.8%) necessitated hospital admission for the required healthcare services before being discharged. Death was reported in only two cases (case fatality rate of 0.1%), as shown in Table 3.

**Tables 3.** Management and outcomes of confirmed dengue fever cases in the period from 22021 to 2022 (*N* = 1977 cases)

Characteristics		No	%
The patient’s treatment received^a^	Antibiotic	1719	86.9
	Antipyretic	1930	97.6
	IV fluid	217	10.9
Outcomes of dengue fever (*N* = 1977)	Receive outpatient services	1881	95.14
	Received inpatient services and then discharged	94	4.8
	Death	2	0.1

a Multiple response analysis.

## Discussion

The aim of the current study is to shed light on Afghanistan's escalating dengue fever cases, most reported symptoms and signs, distribution throughout the country, management measures and death tolls. The total number of reported cases was approximately 2000, with a mortality rate of one per 1000. Pakistan, a neighboring country, reported a higher number of cases (approximately 49 000) over the same full-stop period, yet both countries agreed on a low mortality rate (183 deaths in the latter) [9]. The highest number of reported cases both in Afghanistan and Pakistan could be due to the difficulty in clinically differentiating between dengue fever from one side and COVID-19, Zika and Chikungunya [10]. In addition, the capitals did not report the highest number of cases in both the countries where the burden of disease was much lower compared to other regions which was less than 10% in both countries [9]. The highest burden of disease was within the Nangarhar Province, with almost 98% of cases. All 39 cases reported from Kabul were imported cases from endemic areas (or those who had a travel history to endemic areas), and they were referred to Kabul to seek critical or advanced healthcare services. These figures agree with the dengue epidemic reported in the country in 2019 [4]. There was a steep rise in cases in 2021, with the start of the autumn season in September whereas in 2022, the numbers were higher, with a steep rise from end of September. This could be attributed to an increase in the rainfall that starts to occur in September. In addition, the increase in reported cases can be attributed to the enhanced surveillance system detecting more previously unreported cases. Furthermore, COVID-19 restrictions being lowered combined with improper rubbish disposal, taking into consideration the nature of the disease as vector-borne, could also play important roles [10]. The provided information regarding the epidemic curve underscores the significance of monitoring and addressing dengue outbreaks, particularly during the specified seasonal periods, for effective public health interventions and disease management. Ages 16–25 had the highest number of cases, which tended to decrease as age increased and decreased. The same pattern was observed in other studies [11–13], which could be explained by the fact that this age group is the most active, outgoing and more exposed to mosquito bites. Males represented a higher percentage of cases, which could be explained by being more involved in labor and outdoor occupations, making them at greater risk of exposure to mosquito bites. This is consistent with other studies in neighboring countries, such as Pakistan, Bangladesh, and India [11–14]. A minority of cases reported a travel history to endemic countries, with the disease being present in a minority of cases, which was also noted in neighboring countries [12]. The nature of the disease being arthropod-borne could explain the fact that there is an increasing trend in the number of cases, even though transboundary importation of the virus is minimal. In addition, climate change could have its own effect. The most prevalent symptoms were fever, headache, and muscle pain, the classic mild clinical manifestations of viral hemorrhagic fever, yet there were no reported symptoms of severe manifestations, such as rash and bleeding. This agrees with other studies where the majority of cases reported to have the same mild flu-like viral symptoms without the hemorrhagic severe manifestations [1, 12] less than 5% of cases were hospitalized; that was lower than in Pakistan, 35 cases (5.3%), which recorded a much higher number of reported cases. The diagnosis of dengue fever remains a challenge, as most cases are either underreported and/or undiagnosed [12]. Based on the suspected case definition, any person had an acute onset of fever (>380°C) for 2–10 days with at least two of the following manifestations: a severe headache, retro-orbital pain, myalgia or arthritis, and a positive tourniquet test. In the present study, laboratory investigations were performed. Direct methods were virus rapid detection tests, which were performed for 1086 cases, out of which 1032 were positive (positivity rate of 95%). However, viral PCR was only performed for one-quarter of the patients and was positive in approximately 75%. The former test has recently replaced the latter for the direct method of diagnosis of dengue fever virus infection, which is proven in the current study to be more accurate (95% positivity in RDT compared to 76% in PCR) [15, 16]. Indirect methods, including ElISA, IgG, and IgM, were used in a minority of cases and gave false negative results more than true positives. This could be because samples were collected as early as 1 day and not more than 17 days post-infection, with a low median of 4 days. It is preferred to perform indirect diagnostic tests for dengue fever 7 days after the start of the infection [17].

The limited technical capacity to properly diagnose dengue fever made it difficult to make decisions regarding the proper management of cases and the usage of antibiotics; however, the outcome of DF was satisfactory. Antipyretics were used in 98% of cases and antibiotics in 87%, whereas IV fluids were used in only 11% of cases. This could be attributed to the fact that the majority of clinical manifestations were not severe and had low mortality rates (the case fatality rate was 0.1%). This was obvious from the high rates of discharged patients who received treatment in outpatient clinics (95.14%) and the minority who needed hospitalization for proper medical care and were then discharged.

The current study results should be interpreted with regard to the following limitations: as a retrospective analysis, the data have been collected for surveillance purposes with a descriptive nature, making it susceptible to bias. Since the data for circulating genotyping were not available in this study, it may prevent findings from being extrapolated beyond Afghanistan and other similar low-income countries.

## Conclusion

Afghanistan has witnessed accelerating dengue infection cases. Understanding the epidemiological and clinical characteristics of the suspected reported cases is fundamental to controlling this outbreak and taking strict measures to prevent further spread to and from neighboring endemic countries.

## Supplementary Material

ckae116_Supplementary_Data

## Data Availability

Data will be available upon request.
