# Supplementary material for: Epidemiological pattern of dengue fever in Afghanistan in the period 2021–22
Source: Eur J Public Health. 2025 Jan 13;35(Suppl 1):i23–6. doi: 10.1093/eurpub/ckae116 (PMC11725954; doi:10.1093/eurpub/ckae116)
Supplement: ckae116_Supplementary_Data [file ckae116_supplementary_data.docx]

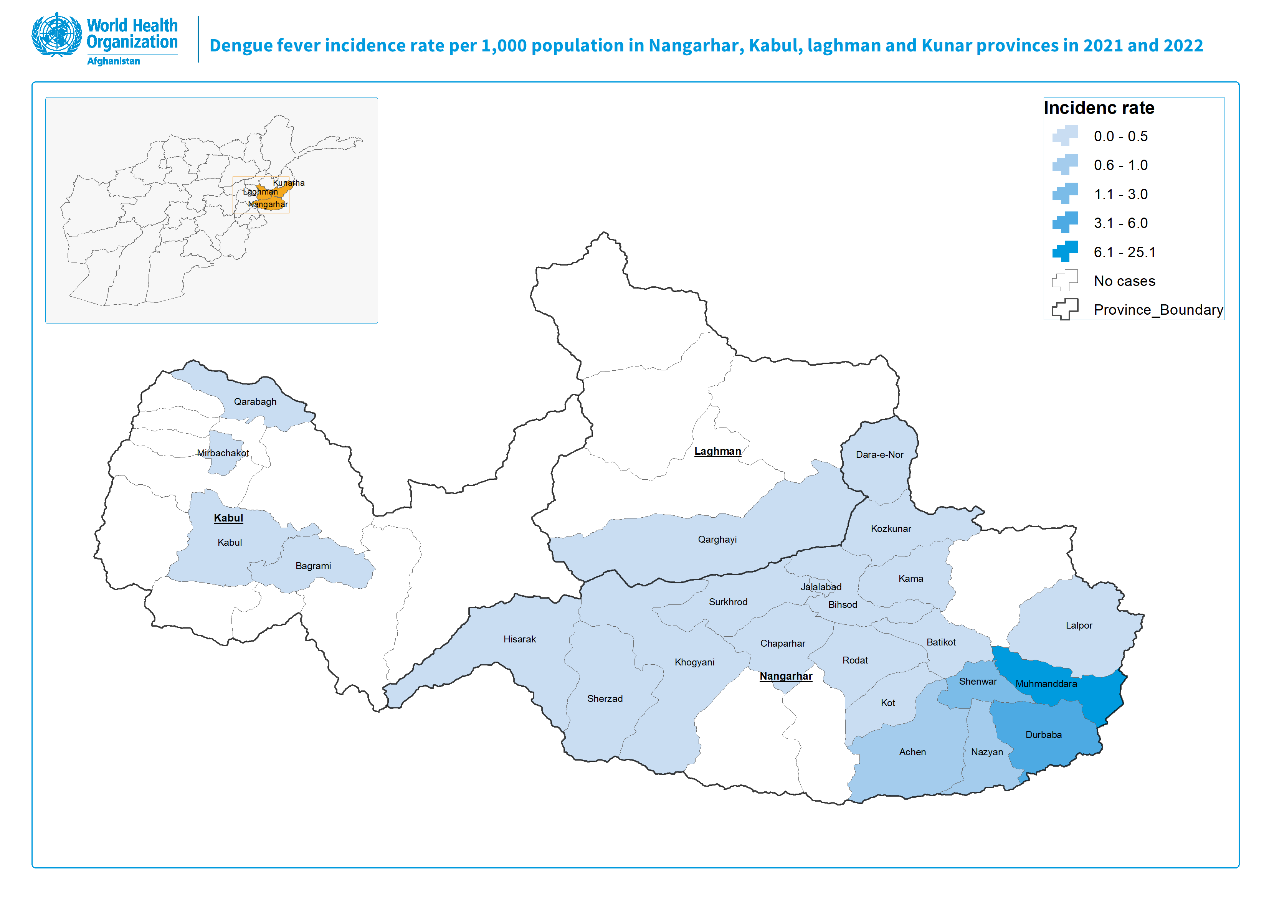


Figure S1 : Map demonstrating the distribution of cases by cumulative incidence and district/province during the dengue fever outbreak in Afghanistan (2021-2022)

**Figure S 2 : The epidemic curve of dengue cases by date of illness onset in Afghanistan during the period from 2021 to 2022**
